# Using Wearable Sensors to Identify Home and Community-Based Movement Using Continuous and Straight Line Stepping Time

**DOI:** 10.3390/s25164979

**Published:** 2025-08-12

**Authors:** Lauren Gracey-McMinn, David Loudon, Alix Chadwell, Samantha Curtin, Chantel Ostler, Malcolm Granat

**Affiliations:** 1 Centre for Human Movement and Rehabilitation, School of Health and Society, University of Salford, Salford M6 6PU, UK; 2 PAL Technologies Ltd., Glasgow G4 0TQ, UK; 3 School of Healthcare Enterprise and Innovation, Faculty of Medicine, University of Southampton, Southampton SO17 1BJ, UK; 4 Portsmouth Hospitals University NHS Trust, Portsmouth PO6 3LY, UK; 5 School of Health Sciences, Faculty of Environment and Life Sciences, University of Southampton, Southampton SO17 1BJ, UK

**Keywords:** community participation, activity classification, accelerometry, activPAL, rehabilitation outcomes, mobility assessment, physical behaviour monitoring, objective measurement

## Abstract

Objective measurement of community participation is essential for evaluating functional recovery and intervention outcomes in clinical populations, yet current methods rely heavily on subjective self-report measures. This study developed and validated a classification model to distinguish between home- and community-based activities using stepping and lying data from activPAL devices. Twenty-four healthy participants wore activPAL 4+ monitors continuously while completing activity diaries over 7 days. A grid search optimisation approach tested threshold combinations for two stepping parameters: straight-line stepping time (SLS) and continuous stepping duration (CSD). The optimal model achieved 93.7% accuracy across 24-h periods using an SLS threshold of 26 s. The model demonstrated high precision with a median difference of just 7 min between the predicted and reported community participation time. Individual variation in model performance highlights the need for validation in diverse clinical cohorts. This represents a methodological advance in objective physical behaviour monitoring, enabling accurate classification of home and community activity from posture data. By identifying not just how much people move but where they move, the model supports more meaningful assessment of functional mobility and community participation. This can enhance clinical decision making, rehabilitation planning, and intervention evaluation. With potential for adoption in clinical pathways and public health policy, this approach addresses a key gap in measuring real-world recovery and independence.

## 1. Introduction

Community participation is a key determinant of both mental and physical health, particularly for populations at risk of isolation, such as older adults [1] and individuals with chronic conditions [2]. In this study, we adopt a spatial definition of community participation, defined as time spent away from the primary residence, consistent with the rehabilitation literature and capturing opportunities for participation in community life and social engagement [3,4]. Limited social engagement has been consistently linked to depression [5] and may contribute to the onset or progression of chronic illnesses over time [6]. Individuals who seldom leave their homes also engage in less physical activity, even when indoor exercise is considered [7,8]. In contrast, community-based activities, such as shopping, commuting, and socialising can promote higher physical activity levels, which are associated with reduced risks of comorbidities such as obesity [9], poor body image [10], impaired balance [11], and depression [12]. This highlights the potential of increased community participation as a preventive and therapeutic strategy across both clinical and ageing populations, but despite these perceived benefits, many clinical populations continue to experience restrictions in their ability to engage with their communities, even following therapeutic or rehabilitative interventions [13,14,15]. Understanding the reasons behind limited community participation remains a challenge, in part due to the lack of consensus on how to best capture community participation, with a range of tools being used inconsistently across studies [4,16] and the difficulty of measuring participation directly [17,18]. Many studies have relied on subjective approaches such as self-reported questionnaires or clinical assessments, which may lack ecological validity [19], are often influenced by individual bias [20], and often fail to capture the dynamic and contextual nature of real-world community participation. While physical, psychosocial, and environmental factors all influence community participation and engagement, their effects vary widely between individuals. As a result, both clinical and self-reported assessments often focus on aspects of physical impairments or activity limitations, rather than directly measuring participation restriction itself [4].

Wearable sensors offer a promising solution, providing the opportunity for objective and continuous long-term monitoring of community participation without the need for patient input. This would offer insights into functional capacity over a sustained period of daily living, beyond the brief and artificial snapshot of a patient’s abilities seen in a clinical setting. Such technology could lower patient burden and provide clinicians and policymakers with actionable data to assess and improve rehabilitation strategies, healthcare and assistive technology provision, and social policy.

Previous research has explored the use of GPS to track community participation [21,22], but while GPS offers location-based insight, these devices are power-intensive, requiring frequent charging, demand complex data processing, and may raise privacy concerns. It also provides only limited information about the type and context of movement within the community, requiring additional sensors for activity-specific information [22,23]. Similarly, fixed-point systems such as door sensors or in-home monitors can detect entry or exit but offer no insight into the duration, nature, or context of activity beyond those fixed locations. For example, a door sensor may register someone stepping outside into their garden as having “left home”, even when no meaningful community participation has occurred. In contrast, analysing stepping behaviours captured through wearable activity monitors could offer a low-power, unobtrusive, and scalable method for assessing community participation. These wearable sensors can be deployed quickly in any setting with minimal participant burden, whilst posing few privacy concerns and remaining suitable for a wide range of users, including clinical populations. If stepping patterns can be shown to reflect meaningful community participation, then this approach would enable continuous, objective tracking of both the ability to participate in community-based activities and other functional outcomes over extended periods in a real-world environment. This would provide a direct, quantifiable measure of real-world community participation, integrating contributing factors into a single, reliable metric. Such a metric could be used across many clinical populations and studies to better understand and support community participation.

In order to develop a classification model from stepping patterns that can differentiate between movement at home and in the community, it is first important to define the concept of “home” and “community”. The term “home”, for the purposes of this paper, represents the primary dwelling where an individual spends the majority of their time and is the place where an individual would be most likely to sleep on any given night. It is characterised by the confined space it offers, which will impact movement patterns due to limitations on the extent of continuous movement possible in any given direction. “Community” activity, meanwhile, captures any duration of time that an individual spends away from their home. Activities under this category could include visits to the residences of friends, family, or acquaintances and also include periods spent on exercise, shopping, or socialising outside of their own home, as well as any form of employment. It can therefore consist of activity that falls within both constrained and unconstrained environments.

The aim of this research is to develop an objective method for measuring community participation using stepping patterns recorded from wearable activity monitors, with the intention of creating an outcome measure for use by healthcare professionals and patients. This objective was achieved by developing an activity classification model capable of distinguishing between home- and community-based activities using stepping data from activPAL wearable devices. The model focuses on identifying primary daily transitions—specifically the first departure from home and final return each day—which serve as reliable indicators of community engagement. This paper presents the data collection methodology, describes the systematic development and optimisation of the classification algorithm using grid search techniques, and reports on model performance across multiple evaluation metrics, including accuracy, F1 score, and transition timing precision.

## 2. Materials and Methods

### 2.1. Participants

A convenience sample of 24 participants was recruited to participate in the study (male = 13; female = 11). This sample size (*n* = 24) aligns with or exceeds sizes commonly used in many free-living sensor validation studies [24,25,26,27]. The mean participant age was 36.0 ± 13.5, the mean height was 173.7 ± 9.0 cm, and the mean weight was 76.4 ± 12.8 kg. All participants declared that they had no injuries or illnesses that may impact their mobility. Two participants were retired, two worked part-time in an office-based job, and 20 worked full-time (17 in office-based jobs, 1 as a veterinary surgeon, 1 as a volunteer at a large sporting event, and 1 in retail). The mean number of days normally worked outside of the house for those in employment was 3.8, with a number of participants having hybrid working patterns, sometimes working from home and at other times from their place of work. All participants gave informed consent, and the study was approved by the University of Salford’s School of Health and Society Research Ethics Panel (ref. 6444). Once consent had been given, they were familiarised with the study protocol before data was collected.

### 2.2. Data Collection

The activPAL 4+ activity monitor was selected to record activity data for this study due to its integrated magnetometer, enabling the measurement of straight-line stepping times (and therefore turning frequency) during individual stepping events, as well as the classification of posture behaviours such as lying, standing, and stepping. Analysing behaviour data determined by the proprietary activPAL algorithms reduces computational demands by minimising reliance on complex accelerometer data processing whilst still providing high-resolution details of activity.

Participants were given an activPAL 4+ device (PAL Technologies Ltd., Glasgow, UK) to wear for seven days on their thigh and asked to take part in their normal daily activities throughout data collection. The activPAL 4+ was affixed to the mid-anterior aspect of their chosen thigh using hypoallergenic tape, following the standard protocol described and illustrated elsewhere [26,28]. Thigh-worn accelerometers are considered the “gold-standard” method for capturing movement data across a 24-h cycle [29]. The recommended activPAL wear time for adequate data collection is 7–14 days to allow for daily variation in activity levels throughout the week [30]. However, for the purposes of this work, the number of occurrences of community and home activity were of more importance than a person’s specific patterns of activity, and therefore the lower limit of 7 days was chosen to ensure adequate data storage and battery life whilst still allowing sufficient time for an adequate number of activity bouts.

An external validation method was required to accurately determine participants’ locations during data collection and enable labelling of sensor data for development and evaluation of the classification model. Various methods have previously been used to establish an individual’s location, including direct observation, GPS, and self-report tools [31,32]. While technologies such as GPS or Bluetooth- or Wi-Fi-based positioning have been explored for location detection, they present several limitations that make them less suitable for this type of study. GPS is often unreliable indoors and in built-up environments, where location data may be missing or imprecise [33,34,35]. Conversely, Bluetooth- and Wi-Fi-based positioning systems are prone to errors outdoors, where infrastructure is sparse or inconsistent [36,37]. Furthermore, these technologies impose significant battery demands on wearable devices, which limits feasible wear time and limits the suitability of such systems for multi-day or long-term monitoring. Therefore, self-reported diaries were selected due to their simplicity in data processing for long-term monitoring compared with other methods, the ability to report reasons for leaving home, and successful use in other studies using activPALs [21,35,38,39].

The participants recorded the start time of any new activity or location change, accompanied by a brief activity description. Although not essential for identifying community-based activity, these descriptions facilitated anomaly detection, such as exercise at home or walking in large indoor environments, and may support future analyses of movement behaviours. Participants were also requested to record any periods when the sensors were removed (e.g., during bathing or sleeping).

### 2.3. Data Processing

The collected data was processed from the thigh-worn sensor using PALanalysis (PAL Technologies Ltd., Glasgow, UK) and exported as “Stepping Bouts” with the GHLA v2.2 algorithm, and thus each period of continuous stepping was classed as a separate event. The activity diaries were digitised and then loaded into Python (version 3.11.3) to allow the sensor data to be labelled as occurring in the home or the community according to the diary classifications.

## 3. Model Development

### 3.1. Feature Extraction

It is expected that short periods of stepping and frequent turning will be prevalent in most indoor locations, both at home and in constrained public environments such as offices, restaurants, and shops, due to limited spatial layouts and regular interactions with furniture, walls, or other people. These physical constraints tend to produce similar movement patterns across various indoor settings. However, by analysing broader behavioural patterns before, during, and after such events, it may be possible to distinguish between environments and determine which restricted areas are community-based, and which represent a person’s primary residence or home.

The differing intent behind an individual’s behaviour at home versus in the community also plays a key role in shaping movement patterns. Activities performed outside the home, such as commuting, shopping, or socialising, often involve longer, more purposeful bouts of movement, including walking in straight lines, riding in vehicles, or walking longer distances uninterrupted. In contrast, behaviour at home typically includes more fragmented and less goal-directed movements, frequent transitions between short activities (standing and stepping), shorter periods of straight-line stepping, and longer durations of sedentary behaviour or rest.

To support the classification of home versus community settings, several features were selected:

- Continuous stepping duration: Longer uninterrupted stepping bouts may indicate purposeful movement through larger environments, common in community settings such as commuting or walking outdoors. In contrast, shorter, fragmented bouts are more typical in constrained home environments.
- Straight-line stepping time: Time spent walking in relatively straight paths is likely longer in open or semi-open community environments (e.g., footpaths, work corridors, and parks) and shorter at home due to frequent turns and obstacles.
- Time spent on transport: Transport usage is an obvious indicator of being in the community, particularly for commutes or social outings. Such periods could not occur when someone is at home.
- Periods of sleeping or lying down: Extended periods of low activity or rest, particularly at night, are typically associated with being at home. These can serve as temporal anchors to help classify other activities relative to the home setting.

### 3.2. Differences in Behaviours at Home Versus in the Community

To determine whether certain durations of continuous stepping duration (CSD) or straight-line stepping (SLS) occur exclusively at home or in the community, we identified the 99th percentile cut-off thresholds for stepping events in each location, based on the labelled data. The 99% threshold was chosen to include the vast majority of events while excluding outliers At home, 99% of CSD events were shorter than 54.7 s, and 99% of SLS events were shorter than 22.9 s. In contrast, community-based activity included CSDs up to 555.0 s and SLS durations up to 248.3 s. While many events of shorter durations occurred across both settings, these results clearly indicate that longer-duration stepping events are highly unlikely to occur at home.

**Figure 1:**
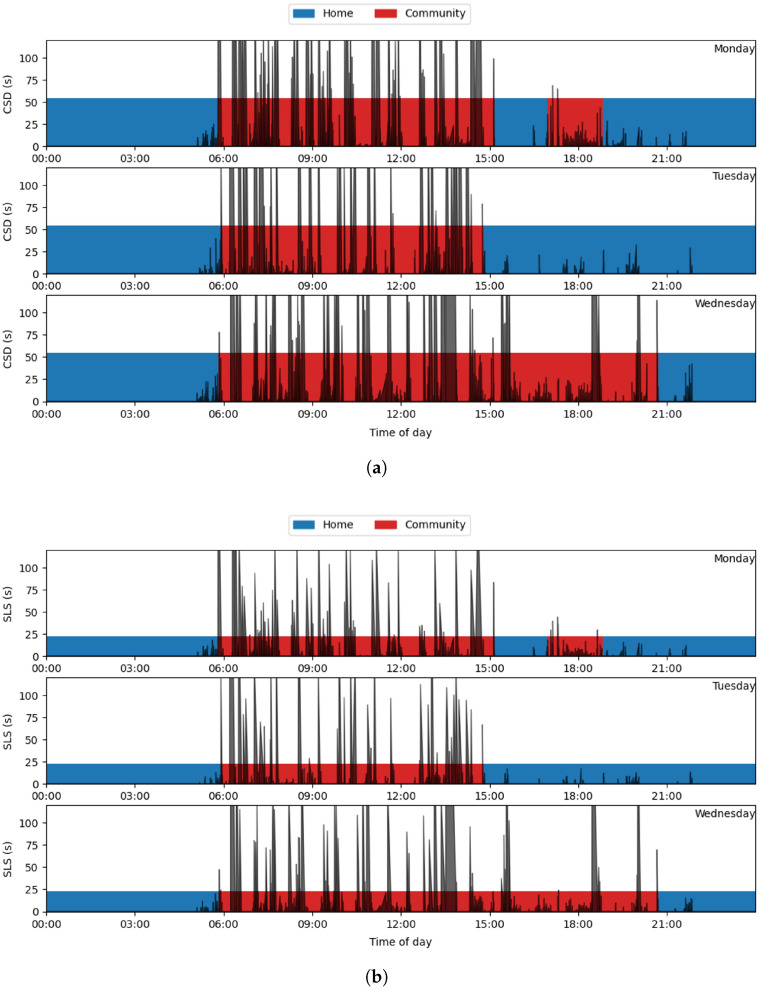
Durations overlaid with self-reported data for (**a**) continuous stepping duration and (**b**) straight line stepping time. The horizontal bars represent the self-reported data, with the bar height equal to the 99% thresholds.

Figure 1 presents an example of CSDs and SLS durations overlaid on a 24-h timeline distinguishing home (blue) and community (red) locations, with 99% threshold values represented by the height of the coloured horizontal bars. The figure illustrates that events exceeding the home-based thresholds were rare at home but common in the community. However, it also reveals a substantial number of shorter-duration stepping events, including those with high turning frequency, occurring outside the home. Therefore, while long-duration stepping events can be used to identify community-based activity, additional methods are needed to detect community activity from events containing shorter-duration bouts.

As well as stepping events, the activPAL 4+ detects periods of lying, separating them into “primary lying” and “secondary lying” through an algorithm that identifies the longest continuous period of predominantly non-upright events each day. Primary lying periods represent the longest period and are assumed to contain the main period of sleep; therefore, these periods are referred to as “sleep” for the purposes of this paper. Secondary lying periods are any additional long periods of lying that are disconnected from the primary lying period, and they are therefore referred to as “lying” for the purposes of this paper. Of 550 total sleeping and lying events, only 9 occurred in the community, and all of these were “secondary lying”. All “primary lying” (sleeping) events occurred whilst the participants were at home. This means that periods of sleeping can be assumed to always occur whilst an individual is at home, whilst “secondary lying” is less reliable and therefore cannot.

Seated transport (e.g., time spent in a car or on a bus or train) cannot occur at home. However, when comparing the times during which seated transport events were identified by the activPAL monitors with self-reported data, it was found that whilst the vast majority (453 out of 569) of seated transport events occurred in the community, there was a notable remainder (20%) that occurred at home. Seated transport is detected by the prorietarty (GHLA) activPAL algorithm via increased noise while in a seated position, but discrepancies occur at times distant from when the participant reported returning home. This suggests that this algorithm may be oversensitive in this instance, possibly recording fidgeting or other repetitive movements as transport, rather than these discrepancies arising from errors in the self-reported data.

Therefore, the features chosen to analyse for the creation of the classification model were the following:

1. Continuous Stepping Duration: The duration of a stepping bout.
2. Straight Line Stepping Time: The longest continuous period during a stepping bout in which the participant’s direction of movement remains relatively straight, defined by a heading change of less than 45°.
3. Sleep: Encompassing periods of sleep (primary lying).

While the features described above can be derived using proprietary analysis algorithms built into the activPAL 4+, they represent general behavioural constructs that are not exclusive to this device. All selected features, including stepping duration, straight-line walking, and periods of sleep, can be identified using alternative activity monitors equipped with an accelerometer and magnetometer, or other sensors capable of capturing movement and orientation. For example, turning can be detected using changes in heading derived from magnetometer data or estimated from gyroscope signals [40,41], while stepping durations and cadence can be extracted from accelerometer-derived gait cycles [42,43]. Similarly, sleep detection has been demonstrated with a variety of wearable sensors [44,45]. Therefore, the underlying principles of this classification method are transferable to other devices, provided that comparable signal features are available.

### 3.3. Identifying Transition Events

While prolonged CSD and SLS durations are rarely observed at home, shorter-duration events also occur outside the home. As such, labeling only longer-duration events as community-based would lead to misclassification of the many shorter bouts that take place in settings like offices, restaurants, or shops—community environments where space constraints limit movement. To address this, prolonged CSD and SLS events can be treated as transitional markers that help contextualise periods of more limited mobility or sedentary behaviour. By leveraging the sequential nature of time series data in this way, this approach enables more accurate classification of stepping patterns by interpreting each activity in relation to its surrounding temporal context.

**Figure 2:**
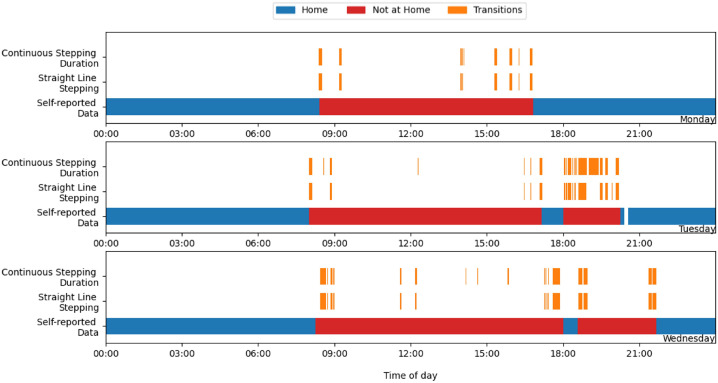
Visualising the locations and durations of potential transition events according to the transition criteria (CSD > 54.7 s, SLS > 22.9 s), with any stepping event meeting the criteria highlighted in orange. Periods of white in the self-reported data symbolise a period of not wearing the activPAL sensor.

Figure 2 highlights the large number of stepping events each day that would meet the criteria for a transition event if using the 99% thresholds defined earlier (CSD > 54.7 s, SLS > 22.9 s). While most of these events occurred within self-reported periods of community activity (as expected), not all represent a movement between a person’s home and the community. In some cases, multiple transition-like events may occur within a single transition period, or several may take place in the same community location as part of sustained engagement with that environment. For instance, a prolonged walking bout may reflect physical activity or intra-location movement rather than travel between distinct places. Transitions may also occur between two non-home locations. To better distinguish between these cases, we defined two categories of transition events:

- Primary transitions: Movements between the participant’s primary location (where they stay overnight) and any other location. They are either leaving or returning home.
- Secondary transitions: Movements between two non-primary locations. This includes extended walking bouts that meet the transition criteria but occur within the context of ongoing community engagement (e.g., walking commutes or moving between shops).

**Figure 3:**
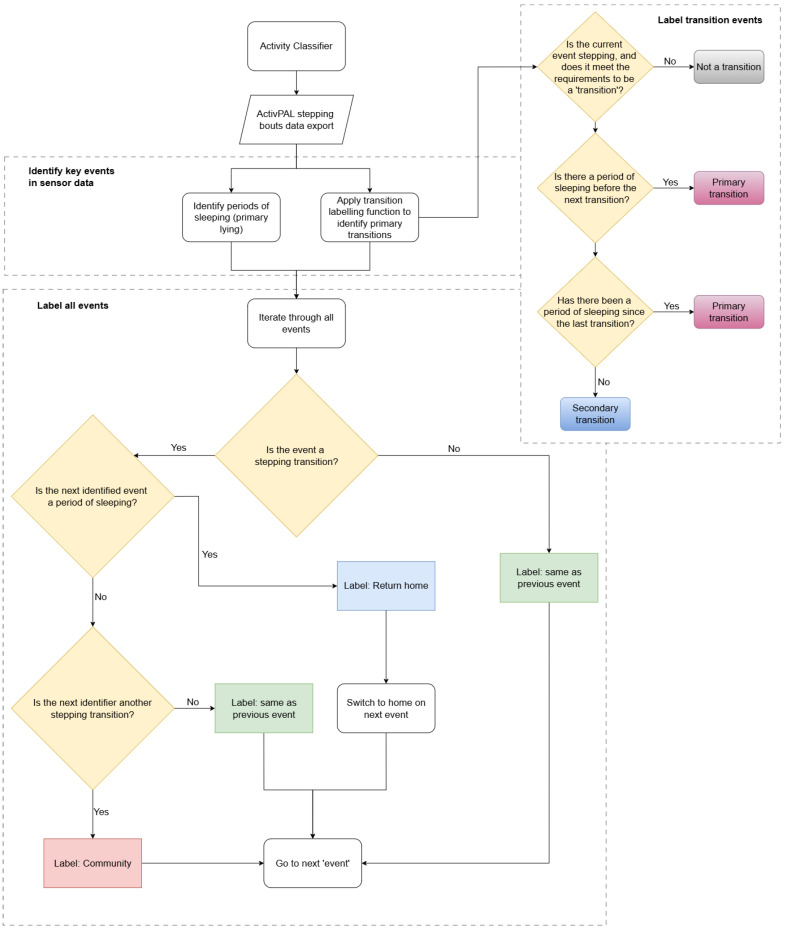
Flow chart for the transition-based community detection algorithm.

To incorporate these transitions into a classification model, we implemented the stepwise process summarised in Figure 3. First, all periods of sleep were identified and labelled. Next, all events meeting the transition criteria were identified and classified as either primary or secondary transitions as described above. Finally, any time interval between two primary transitions that included a period of sleep was classified as home, while all other time was classified as community. The beginning of a leave transition marked an immediate reclassification from home to community, and the end of a return transition triggered an instant reclassification back to home.

**Figure 4:**
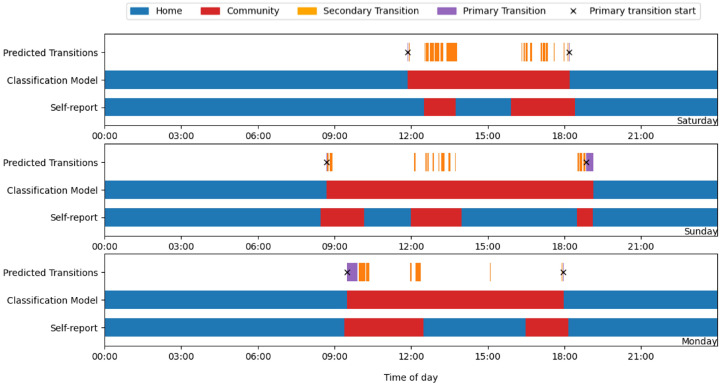
The classifier consistently misidentified activity at home as occurring in the community when participants returned home during the day, as there was no period of sleep.

Figure 4 shows a key limitation with this method, as only periods of sleep were determined to definitely occur at the primary locus, if a participant returned home in the middle of the day before leaving again, the classification model could not identify this. To account for this, and for the purpose of conducting a sensitivity analysis to enable optimisation of critical thresholds, the diary data was “bookended”. This involved ignoring any self-reported returns home during the day and treating all time between the first departure from home and the final return that day as community-based activity in the reference data. Because the participants rarely slept during these brief returns, and the model depends on sleep to confirm one’s presence at home, such periods were inherently misclassified. By bookending the data in this way, we introduced a controlled simplification of the reference data that allowed for a more consistent evaluation and optimisation of transition thresholds, avoiding the variability introduced by unidentifiable midday home periods, which would otherwise bias the results.

### 3.4. Sensitivity Analysis and Threshold Optimisation

A grid search was used to identify the optimal combination of thresholds for each parameter [46]. For both CSD and SLS durations, thresholds were tested across a range from 0 to 200 s initially at 10-s intervals, and then a smaller defined range based on the initial results at 1-s intervals, resulting in a comprehensive grid of possible threshold pairings. Due to the relatively small sample size (*n* = 24) and the high variability between participants, the grid search was conducted using the full dataset rather than separate training and testing splits.

Four different methods of combining the parameters were tested to determine the impact of each parameter, both individually and together, on the output:

- CSD only;
- SLS only;
- CSD or SLS;
- CSD and SLS.

For each combination of thresholds, the algorithm’s prediction of a home or community location was evaluated against the self-reported labels provided by the participants. For the purpose of this analysis, the self-reported data was treated as the ground truth. This approach allowed for the identification of threshold values that yielded the highest model performance when compared with participants’ self-reporting.

To evaluate the performance of the classification model and determine the optimal threshold values for CSDs and SLS durations, multiple performance metrics were calculated. These were accuracy, F1 score, sensitivity, specificity, and precision. Accuracy and F1 score were used as the primary ranking criteria for threshold optimisation. Accuracy measures the proportion of correct predictions across all classes (in this case, all events (including stepping, lying, sitting, and standing) whether at home or in the community), providing a general sense of model performance. However, in scenarios where the distribution of classes (home and community) is imbalanced or where false positives and false negatives carry different consequences, accuracy can be misleading without additional analysis, as it may be biased toward the majority class [47]. The F1 score, as the harmonic mean of precision and recall, offers a more informative measure in such scenarios by ensuring that the performance for both classes is adequately represented [48,49]. The accuracy and F1 scores were calculated per day of recorded data, with the median result taken to limit the impact of outliers and individual participant bias on the results. Individual participant bias is particularly important to consider in activity classification models, as the participants may have had vastly different mobility patterns, living arrangements, or adherence to the study protocol, which could lead to systematically higher or lower classification performance for certain individuals. While the dataset contained a substantial number of datapoints (120,649), the relatively small number of participants (24) meant that extreme values from individual participants could disproportionately influence mean-based summary statistics.

## 4. Results

### 4.1. Classification Model Performance

The optimal thresholds found during the grid search for each logic type are shown and ranked in Table 1. Optimal thresholds were chosen based on the highest F1 score and then accuracy, with ties broken by the lowest threshold value. All combinations produced strong classification performance, with all approaches except CSD-only achieving over 90% accuracy during waking hours. The CSD-only logic reached a slightly lower accuracy of 88.5%, suggesting that CSD is the less informative of the two parameters.

Several findings support SLS as the more valuable parameter for community detection. First, the SLS-only model achieved strong performance (92.9% F1 score), matching the performance of combined approaches. Second, the optimal CSD and SLS thresholds included a CSD value of 0 s, effectively making this equivalent to SLS-only classification. Third, when using CSD-or-SLS logic, the optimal CSD threshold increased substantially to 95 s, compared with 77 s for the CSD-only approach, suggesting that the SLS threshold (26 s) is typically met first, making the CSD component redundant.

**Figure 5:**
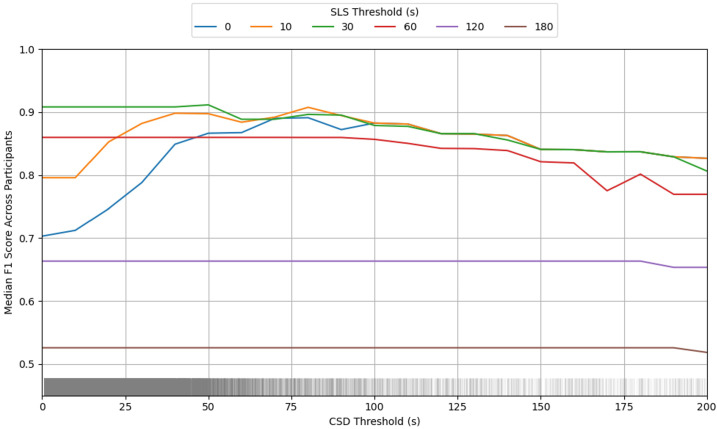
Interaction plot showing how the F1 score alters with different combinations of CSD and SLS thresholds when the stepping event must meet both of the threshold values. The bars along the bottom of the plot show the prevalence of events by CSD.

The interaction plot in Figure 5 further illustrates this relationship, showing that CSD threshold values only meaningfully impact F1 scores when SLS thresholds are very low (0–10 s). At higher SLS thresholds, the F1 scores remained relatively stable across CSD values before eventually decreasing at very high CSD thresholds. This pattern confirms that SLS is the primary driver of classification performance, and suggests that using SLS alone could reduce computational complexity without compromising model performance.

Despite SLS being the stronger parameter, the CSD-only model still achieved 88.5% accuracy, indicating that CSD can provide reliable classification when SLS time is unavailable, such as when using older activPAL sensors without magnetometers that cannot detect turning events. All logic-types demonstrated robust performance at their optimal values, suggesting good generalisability across individuals.

When the optimal thresholds identified from the bookended analysis were applied to the original self-reported data (including both mid-day transitions and periods of sleep), the results showed a notable shift in performance patterns (Table 2). Most importantly, the CSD-only logic achieved a higher accuracy (94.7%) compared with the SLS-only approach (93.7%), reversing the performance hierarchy observed in the bookended analysis.

It is important to emphasise that the bookended approach remained the most appropriate method for threshold optimisation in this context. The bookended analysis eliminated the confounding effect of mid-day transitions, which the model cannot reliably detect due to the similar activity patterns that may occur in both home and community environments during the middle of the day. By focusing on the clear behavioural transitions—when people definitively leave home for the first time and return home for the last time each day—the bookended approach provided the most accurate assessment of the model’s ability to identify these critical transition points, which is the primary objective of this classification system. The performance differences observed when applying these thresholds to the full dataset highlight the complexity of location classification and reinforce why the bookended approach was necessary for robust threshold optimisation. The bookended analysis specifically focused on identifying the precise timing of the first departure from home and the final return home. For that reason, the remaining analysis used the classification model with SLS-only logic and a threshold of 26 s.

**Figure 6:**
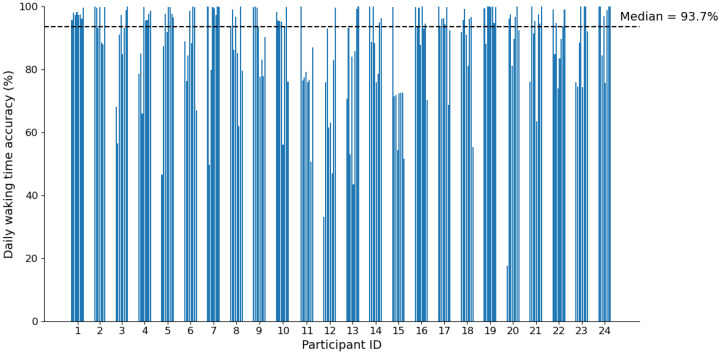
Daily accuracy of the classification model evaluated on the original self-reported data, separated by participant. Each bar represents one day. Accuracy is not consistent across days or participants.

The optimised classifier (SLS-only, 26 s) achieved a median daily accuracy of 93.7% when applied to the full 24-h dataset (Table 2). While this strong overall performance demonstrates the model’s effectiveness, there was notable variation between individuals, with participants 11, 12, and 15 exhibiting consistently lower daily accuracy across multiple days (Figure 6). This inter-individual variation suggests that despite efforts to develop a generalisable model, performance may be influenced by participant-specific factors such as differences in daily routines, activity patterns, or mobility behaviours. For instance, participants with lower accuracy scores may have frequently used seated transport modes, which were excluded from model development. Additionally, some participants may have had more complex daily movement patterns, with frequent brief excursions from home, making it challenging for the model to accurately classify mid-day location changes. It is important to note that much of the classification errors likely stem from the model’s inherent limitation in detecting mid-day transitions rather than errors in identifying the primary departure and return transitions that the model was specifically optimised to detect.

### 4.2. Time Between First and Last Transitions

Beyond overall classification accuracy, the precision of transition timing is particularly important for practical applications of this model. The time in between the first and last transition of each day could provide an insight into the ability of a person to access their community, with longer durations between first leaving and last returning home suggesting a better physical ability due to either leaving and returning home multiple times during a day, or remaining out for extended periods. In contrast, shorter durations may suggest reduced physical ability, fatigue, or a lack of motivation or opportunity to remain active in the community beyond essential tasks.

**Figure 7:**
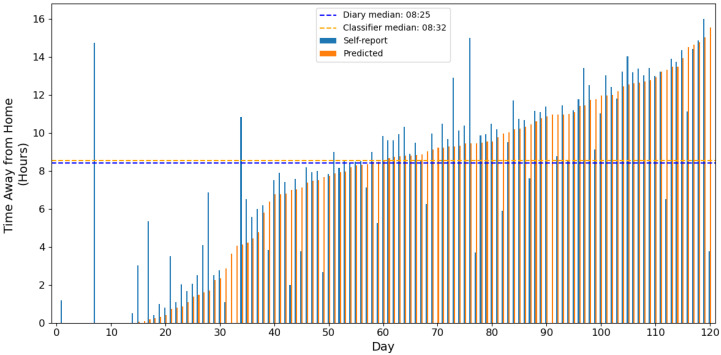
Time away from home for each day of recorded data with more than 12 h, according to the bookended self-reported data and the classification model (predicted).

Given the clinical significance of this measure, accurate detection of these key daily transitions is essential. Figure 7 demonstrates the model’s performance in capturing these critical time points, showing that the daily median time away from home was 8 h and 32 min according to self-reports, compared with the 8 h and 25 min predicted by the model, a difference of just 7 min. This close alignment between the predicted and reported values indicates that the model successfully identified the timing of the first departure and final return home with high precision.

While some outliers were present, where the predicted times deviated substantially from the reported times, these likely represent days when the model either missed a genuine transition or incorrectly identified a false transition. However, the overall pattern demonstrates robust performance, with the median absolute difference between the predicted and reported time away from home being just 40.1 min across all participant days. Considering that this encompassed potential errors in both the first departure and final return transitions, this suggests an average timing accuracy of approximately 20 min per transition, a level of precision that includes the influence of recall bias in the participants’ self-reporting.

These results confirm that the optimised SLS-based classifier can reliably quantify daily community participation durations by accurately detecting when individuals first leave home and when they make their final returns each day, providing a valuable objective measure for assessing community engagement patterns.

### 4.3. Day Detection

Building on the model’s ability to detect individual transitions, a broader application involves determining whether a patient accessed their community on any given day, a binary classification that can serve as a valuable tool for monitoring functional recovery and community engagement, particularly for individuals with significant health challenges. For this analysis, a community day was defined as any day in which at least one primary transition occurred (indicating movement between home and the community), while a home day was defined as a day with no primary transitions detected.

The classification model achieved a high overall accuracy of 94.8% in distinguishing between home and community days. However, the performance varied considerably between the two categories due to the highly imbalanced nature of the dataset. Of the 155 total participant-days analysed, only 17 were true home-only days, and these represented the most challenging cases for the classifier. Specifically, 5 of the 17 home days (29.4%) were incorrectly classified as community days, while only 3 of the 138 community days (2.2%) were misclassified as home days.

This imbalanced performance likely reflects both the nature of the participant sample and the inherent difficulty in detecting the absence of activity. The limited number of home-only days stems from the demographic characteristics of this pilot study population; none of the 24 participants reported illness or injury affecting mobility, and the majority (20 out of 24) were in full-time employment. Consequently, most participants maintained regular community participation patterns throughout the study period. However, the intended clinical application of this classification model involves populations where individuals are more likely to experience mobility limitations and may spend greater proportions of time at home due to illness, rehabilitation, or reduced work capacity. In such populations, the proportion of home-only days would be substantially higher, potentially improving the model’s ability to learn and accurately classify these patterns. While the model demonstrated strong overall performance in this pilot sample, its accuracy specifically for home-only days may not yet be fully representative of real-world clinical applications where such days are more prevalent.

## 5. Discussion and Conclusions

This study presents the development and validation of an activity classification model capable of distinguishing between home- and community-based activity with high accuracy across 24-h periods. Through systematic threshold optimisation using a grid search approach, the optimal model was identified as using straight-line stepping (SLS) time with a 26-s threshold to detect walking bouts that occur outside the home environment. This model achieved a 92.9% F1 score and 91.8% accuracy when evaluated against the critical first departure and final return home transitions each day and 93.7% accuracy when applied to full 24-h datasets.

The model’s strength lies in its focus on primary transitions, namely movements between home and community that are anchored by sleep periods at home. By leveraging the sequential nature of time series data and participants’ natural sleep-wake cycles, the algorithm reliably identifies when individuals first leave home and when they make their final return each day. However, this approach has inherent limitations; the model does not detect brief mid-day returns home that do not involve sleep periods, which likely contributed to some classification errors observed in the full dataset analysis.

The threshold optimisation revealed important insights about the relative value of different stepping parameters. While both straight-line stepping time and continuous stepping duration proved effective for classification, SLS emerged as the superior parameter. This was evidenced by the strong performance of the SLS-only model and the finding that optimal combined thresholds effectively reduced to SLS-only classification (with CSD thresholds of 0 s). The interaction analysis further demonstrated that CSD has a meaningful impact on performance only when the SLS thresholds are extremely low, confirming SLS as the primary driver of classification accuracy. Nevertheless, the CSD-only approach achieved 88.5% accuracy, making it a viable alternative when magnetometer data is unavailable in older activPAL devices.

### 5.1. Clinical Applications and Validation Considerations

The model demonstrated particular strength in detecting community access days, achieving 94.8% overall accuracy in distinguishing between days when participants accessed the community versus staying home entirely. However, performance was notably imbalanced due to the healthy, employed study population; while only 2.2% of community days were misclassified, 29.4% of home-only days were incorrectly identified. This reflects both the rarity of home-only days in this active population (17 out of 155 total days) and the inherent challenge of detecting the absence of community activity.

For transition timing precision—a critical measure for clinical applications—the model demonstrated remarkable accuracy. The median difference between the predicted and self-reported total time away from home was just 7 min. This level of precision, which accounts for potential errors in both departure and return transitions, and likely self-report errors, suggests that the model can reliably quantify daily community participation durations within clinically meaningful time frames.

### 5.2. Limitations and Future Directions

Several important limitations must be acknowledged. The model was developed using data from 24 able-bodied, healthy participants, most of whom were in full-time employment. This population exhibited consistently high community participation, which may not reflect the activity patterns of clinical populations with mobility limitations, chronic conditions, or reduced work capacities. The low prevalence of home-only days in this sample means the model’s performance on such days may not generalise to clinical settings where extended home periods are more common.

While the sample size is typical for early-stage free-living validation studies and adequate for establishing feasibility, a conventional train and test split was not implemented because participants exhibited substantial day-to-day variation in activity patterns. This included differences in commuting methods and durations, employment type (sedentary versus physically active), hybrid working routines, and frequency of brief excursions from home. With a small sample, splitting the dataset risked reducing the representation of this behavioural diversity in either subset, potentially destabilising threshold optimisation. Larger and more heterogeneous samples in future work will allow the use of dedicated training and testing splits or cross-subject validation to formally confirm model performance across clinical populations and real-world variability.

This lack of cross-subject validation or use of a dedicated testing set means the reported model performance may reflect some degree of overfitting. Notably, individual variation in model performance was observed, with some participants showing consistently lower daily accuracies. This suggests that, despite efforts to develop a generalisable approach, participant-specific factors such as unique mobility patterns, frequent use of seated transport, or complex daily routines may influence the model’s effectiveness. Additional challenges may arise when applying this model to less mobile clinical populations who rely heavily on seated transport for community access. In this study, activPAL-detected seated transport events were excluded from the classification features due to the tendency of the proprietary analysis algorithms to detect vehicular movement even when participants were stationary at home, which would have introduced false detection of community activity. For clinical cohorts, it may be necessary to include seated transport data and adjust classification thresholds to better reflect different patterns of mobility and community access.

Future research should therefore focus on validating the model in larger, more diverse clinical cohorts and exploring approaches to improve detection of mid-day home periods and accommodate individual behavioural patterns. Additionally, training and testing splits or cross-validation should be implemented to better evaluate model generalisability. As some of the observed misclassification may be attributable to inaccuracies or self-reporting bias in activity diaries, future studies may also consider incorporating GPS or other location-sensing technologies as supplementary validation tools to help reduce ground-truth uncertainty.

### 5.3. Broader Impact and Applications

This classification model offers several valuable applications for clinical practice and research. The ability to objectively quantify community access days provides a novel outcome measure for rehabilitation and intervention studies. The precise timing of daily transitions enables calculation of community participation duration, offering insights into functional capacity and recovery progress. Furthermore, because the model utilises stepping data from activity monitors, it enables comprehensive analysis of in-community physical activity patterns, including step count, cadence, and other mobility metrics that provide additional context about participants’ physical capacity while engaged in community activities.

The model’s potential extends beyond individual monitoring to intervention evaluation. As demonstrated in previous research, where an early version of this model was used alongside prosthesis wear time algorithms to assess prosthetic interventions [50], this approach can support comprehensive evaluation of clinical outcomes. Such integrated approaches could inform evidence-based clinical decision making and contribute to the development of more effective rehabilitation strategies.

Although this study used features derived from the activPAL 4+ activity monitor, the classification approach is based on behavioural patterns that are device-agnostic. Future implementations could apply similar logic using other wearable devices equipped with inertial sensors, making the method widely applicable across research and clinical settings.

In conclusion, this study developed a robust, computationally efficient classification model that provides objective, quantitative measures of community participation patterns. With 94% accuracy in distinguishing home and community activity and precise transition timing detection, this model represents a valuable tool for clinical assessment, intervention evaluation, and research into community participation patterns. Importantly, the use of a wearable activity monitor enables the simultaneous collection of traditional physical activity metrics such as step count, sedentary time, and postural transitions without increasing participant or patient burden. This integration enhances the practicality and scalability of the approach for both clinical and research applications. Future validation in diverse clinical populations will be essential to realise its full potential for informing clinical practice, public health policy, and the design of more accessible community environments.

## Figures and Tables

**Table 1:** Top performing CSD and SLS thresholds for each logic type with “bookended” self-reported data and only during waking periods.

Logic	CSD Threshold	SLS Threshold	F1 Score	Accuracy	Sensitivity	Precision	Specificity
SLS only	0	26	0.929	0.918	0.933	0.964	0.956
CSD only	77	0	0.900	0.885	0.854	0.950	0.931
CSD or SLS	95	26	0.920	0.902	0.933	0.954	0.906
CSD and SLS	0	26	0.929	0.918	0.933	0.964	0.956

Thresholds refer to optimal values based on the highest F1 score, with ties broken by the lowest threshold value.

**Table 2:** Top performing CSD and SLS thresholds for each logic type on the original self-reported data over 24-h periods.

Logic	CSD Threshold	SLS Threshold	Accuracy	F1 Score	Sensitivity	Specificity	Precision
SLS only	0	26	0.937	0.815	0.936	0.997	0.868
CSD only	77	0	0.947	0.816	0.911	1.000	0.879
CSD or SLS	95	26	0.936	0.811	0.964	0.991	0.819
CSD and SLS	0	26	0.937	0.815	0.936	0.997	0.868

## Data Availability

The raw data supporting the conclusions of this article will be made available by the authors on request.

## Abbreviations

The following abbreviations are used in this manuscript:

CSD	Continuous stepping duration
SLS	Straight-line stepping
